# The *Carthamus tinctorius* L. genome sequence provides insights into synthesis of unsaturated fatty acids

**DOI:** 10.1186/s12864-024-10405-z

**Published:** 2024-05-23

**Authors:** Yuanyuan Dong, Xiaojie Wang, Naveed Ahmad, Yepeng Sun, Yuanxin Wang, Xiuming Liu, Na Yao, Yang Jing, Linna Du, Xiaowei Li, Nan Wang, Weican Liu, Fawei Wang, Xiaokun Li, Haiyan Li

**Affiliations:** 1 Engineering Research Center of Bioreactor and Pharmaceutical Development, College of Life Sciences, Ministry of Education, Jilin Agricultural University, Changchun, 130118 China; 2 School of Pharmaceutical Science, Key Laboratory of Biotechnology and Pharmaceutical Engineering of Zhejiang Province, Wenzhou Medical University, Wenzhou, 325035 China; 3 Sanya Nanfan Research Institute of Hainan University, Sanya, 572025 China

**Keywords:** Safflower, Whole genome duplication, Evolutionary history, Fatty acid biosynthesis

## Abstract

Domesticated safflower (*Carthamus tinctorius* L.) is a widely cultivated edible oil crop. However, despite its economic importance, the genetic basis underlying key traits such as oil content, resistance to biotic and abiotic stresses, and flowering time remains poorly understood. Here, we present the genome assembly for *C. tinctorius* variety *Jihong01*, which was obtained by integrating Oxford Nanopore Technologies (ONT) and BGI-SEQ500 sequencing results. The assembled genome was 1,061.1 Mb, and consisted of 32,379 protein-coding genes, 97.71% of which were functionally annotated. Safflower had a recent whole genome duplication (WGD) event in evolution history and diverged from sunflower approximately 37.3 million years ago. Through comparative genomic analysis at five seed development stages, we unveiled the pivotal roles of fatty acid desaturase 2 (FAD2) and fatty acid desaturase 6 (FAD6) in linoleic acid (LA) biosynthesis. Similarly, the differential gene expression analysis further reinforced the significance of these genes in regulating LA accumulation. Moreover, our investigation of seed fatty acid composition at different seed developmental stages unveiled the crucial roles of FAD2 and FAD6 in LA biosynthesis. These findings offer important insights into enhancing breeding programs for the improvement of quality traits and provide reference resource for further research on the natural properties of safflower.

**Supplementary Information:**

The online version contains supplementary material available at 10.1186/s12864-024-10405-z.

## Introduction

Safflower (*Carthamus tinctorius* L.) is a diploid (2n = 24) dicot plant in the family Asteraceae (Compositae) [1]. It is an annual plant that is predominantly self-pollinated. This herbaceous crop is adapted to hot and dry environments due to its deep root system and xerophytic spines. Therefore, it is widely cultivated in arid and semiarid regions [2]. Safflower is assumed to have been domesticated in the Fertile Cresent region over 4,000 years ago, and it has a long history of cultivation in Asia, the Mediterranean region, Europe, and the Americas [3–5].

Safflower is mainly grown as an oil crop, it has been cultivated for use as birdseed and as a source of oil for the paint industry [6, 7]. In some areas such as Western Europe, safflower is cultivated as a source of Safflor Yellow (SY) that is produced in the floret, and used as a natural dyestuff [8]. Safflower is valuable as an edible oil crop because it produces a large amount of oil (approx. 25% oil content in seeds). It has relatively higher polyunsaturated/saturated ratios than other edible oil, which is rich in octadecadienoic acid and contains more than 70% LA [9]. As a type of essential polyunsaturated fatty acid (PUFA), LA is vital in the dietary composition for both humans and animals. As one of the oldest sources of oil for humans worldwide, the main economic traits of cultivated safflower varieties are related to its composition proportion in LA [10, 11]. Fatty acids desaturase such as FAD2 play a crucial role in regulating the composition of fatty acids, including LA. These enzymes catalyze the desaturation reactions necessary for the synthesis of unsaturated fatty acids, from saturated or monounsaturated precursors [12]. Although, it is not a mainstream oilseed crop in today’s world, it has been cultivated widely and distributed across various geographic regions. The species diversity of safflower could also serve as an important resource for genetic breeding.

The genetic diversity and natural variations in safflower have been studied using several molecular and analytical methods in recent decades [3, 13]. More recent studies have provided molecular information for safflower including its complete chloroplast genome [14], full-length transcriptome [15], and the locations of 2,008,196 single nucleotide polymorphisms, which were identified from recombinant inbred safflower lines [16]. The results of those studies and others indicated that the genetic architecture and evolution of safflower domestication are complex [17]. Such complexity has posed challenges to safflower breeding endeavors. In the past, breeding programs have used hybridization to breed new cultivars [18] and have characterized the safflower germplasm using various molecular markers, including expressed sequence tags, inter simple sequence repeats, single nucleotide polymorphism, and simple sequence repeat markers [15, 19–22]. Genome evolution involves intricate mechanisms such as gene duplication, divergence, and selection, which shape the genetic landscape of organisms over time. Gene duplication, in particular, serves as a significant driver of genome evolution, often leading to the expansion of gene families [23, 24]. Therefore, high-quality safflower reference genome and genome evolution research can reveal genetic structure and phylogenetic details, as well as biosynthesis processes of bioactive compounds. The inaugural sequencing of the safflower cultivar Anhui-1 genome employed PacBio Sequel (Pacific Biosciences) in conjunction with the Illumina Hiseq 2500 sequencing platform. The investigation primarily targeted the biosynthetic pathways of hydroxysafflor yellow A and unsaturated fatty acid [25].

To deepen our understanding of the genetic landscape of safflower, we conducted genome assembly of the Jihong01 safflower cultivar. This particular landrace is extensively cultivated and sourced from western China. It is also used as a main source of breeding novel safflower varieties with improved medicinal properties. In this research, we provide a genome overview of safflower that includes details of genome evolution, gene family expansion, and putative genes for unsaturated fatty acids biosynthesis and their composition. This reference genome will serve as a platform for investigating the genome background, and for identifying important genes to exploit in genetic breeding programs.

## Results

### Genome sequencing and assembly

**Fig. 1:**
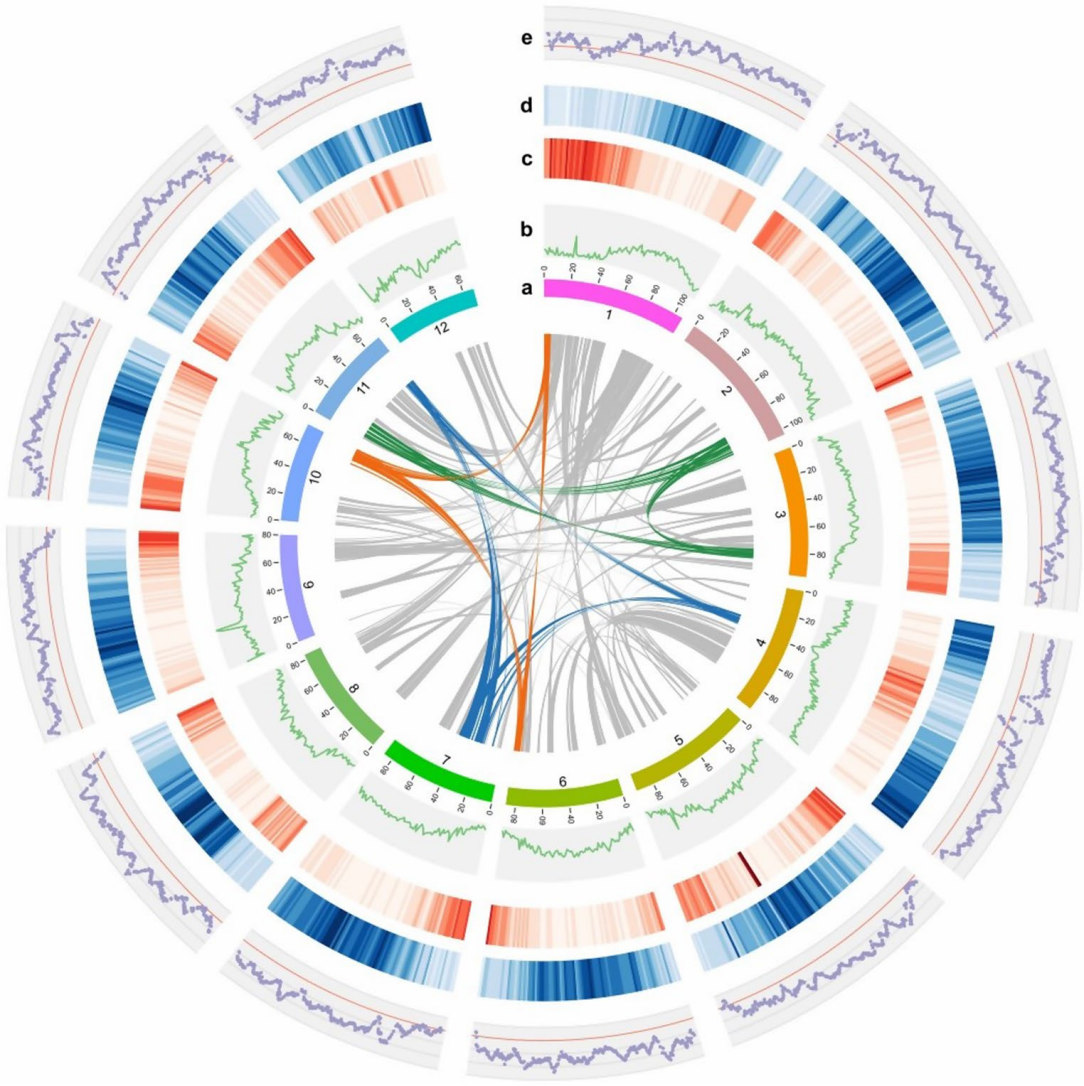
Overview of the *Carthamus tinctorius* genome. (**a**) Chromosomal pseudomolecules. (**b**) GC content (1 Mb windows). (**c**) Gene density (1 Mb windows). (**d**) TE density (1 Mb windows). (**e**) LAI score (3 Mb windows with 300 Kb sliding step). Inner grey ribbons indicate links of synteny blocks, while colored ribbons highlight the residues of whole genome triplication

Genomic DNA was extracted from leaves of the “*Jihong01*” safflower variety and sequenced on BGI-SEQ500 and Oxford Nanopore Technologies (ONT) platforms. We obtained 101 Gb short reads and 130 Gb long reads data in total. By a 17-mer frequency statistics, the size of safflower genome was estimated to be 1,061.1 Mb (Figure S1). The primary contigs was assembled by NECAT software (https://github.com/xiaochuanle/NECAT) with an N50 of 8.6 Mb. The initial assembly was error-corrected by Pilon [26], and the redundant sequences were removed by HaploMerger2 [27]. After several steps of polishing, the total length of the final assembly was 1,061.1 Mb which is close to the estimated genome size (Table S1). To generate a chromosomal-level assembly of the safflower genome, a high-throughput chromosome conformation capture (Hi-C) library was constructed and produced 51 Gb valid reads (47×). 98.82% of the final assembly was anchored to 12 pseudochromosomes with length ranging from 68.06 Mb to 106.66 Mb. GC content of the final assembly was 38.37%. The completeness of our assembly was evaluated by BUSCO (Benchmarking Universal Single Copy Orthologs) [28]. In embryophyta_odb9 dataset, 90.3% BUSCO genes was complete, 2.4% fragmented and 7.3% missing in the assembly. Also, we used LTR Assembly Index (LAI) [29] values to evaluate the quality of non-coding region in the assembly (Fig. 1). The LAI score of our assembly was 21.94, suggesting a high-quality safflower genome assembly.

### Genome annotation

We identified 63.4% of the assembly as repetitive sequences. The proportion is comparatively close to artichoke (58.4%) [30]but lower than sunflower (74.7%) [31] and lettuce (74.2%) [32], which may be the reason why the genome size of lettuce or sunflower is two to three times larger than that of artichoke and safflower [33]. The most abundant transposable elements (TEs) were long terminal repeat (LTR) retrotransposons, accounting for 54.2% of the assembly. Like most plants, *Gypsy* (26.9%) and *Copia* (25.2%) were found to be the two dominant LTR super families. Similarly, insertion time of LTR was estimated at 1.5 Mya based on the sequence divergence of all LTRs, later than artichoke (Figure S2). Importantly, the DNA transposons covered 7.1% of the assembly. A total of 32,379 protein-coding genes were predicted using a combination of homology prediction and transcripts supporting. Distributions of gene set parameters showed a consistent trend with other plants (Figure S3). The gene set covered 93.9% complete BUSCOs of embryophyte BUSCO groups. A sum of 97.71% of predicted proteins were functionally annotated against public protein databases (InterPro, UniProt and KEGG). Besides protein-coding genes, we also annotated 131 miRNAs, 998 tRNAs, 3,017 rRNAs and 1,408 snRNAs.

### Gene family and phylogeny analysis

**Fig. 2:**
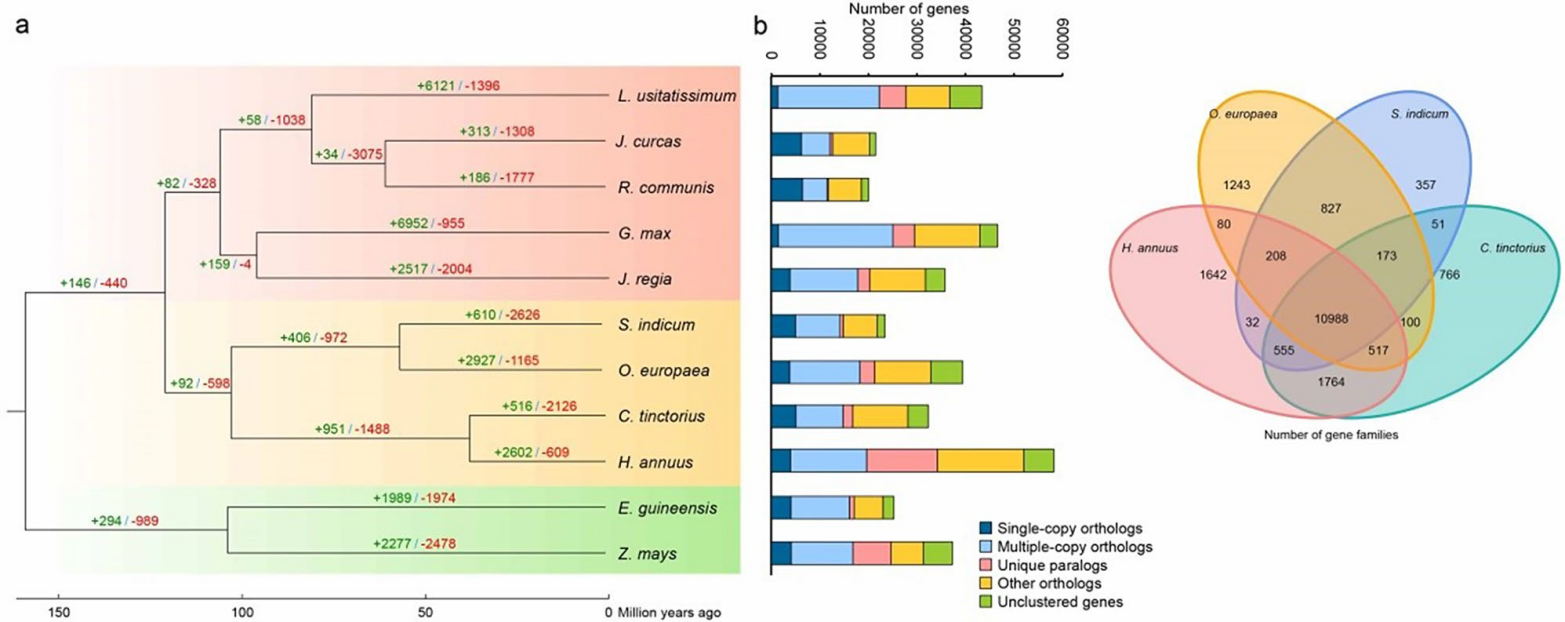
Comparative analysis of *Carthamus tinctorius* with other oil crops. (**a**) Phylogeny, divergence time and gene family expansion/contraction of 11 species. The green numbers are families under size expansion while the red numbers are families under size contraction. The vertical stacked column right is the ortholog genes in 11 species. (**b**) Venn diagram of safflower, sesame, olive tree and sunflower

Phylogenetic tree was reconstructed based on the coding sequences (CDS) of 212 single-copy gene families. Plants of Monocotyledons, Rosidae and Asteridae were separated into respective branches and each species were clustered at the reported evolutionary positions. Noticeably, safflower and sunflower were clustered into one branch in the phylogenetic tree (Fig. 2a). Divergence time of safflower and sunflower was estimated at approximately 37.3 Mya, after the whole genome duplication event at the basal of Asteraceae family [34]. For comparative analysis, we chose high-quality proteins of 10 oil plant species including (oil palm: *Elaeis guineensis*, soybean: *Glycine max*, sunflower: *Helianthus annuus*, Jatropha: *Jatropha curcas*, walnut: *Juglans regia*, flax; *Linum usitatissimum*, olive tree: *Olea europaea*, castor: *Ricinus communis*, sesame: *Sesamum indicum* and maize: *Zea mays*) together with our predicted proteins. All proteins were clustered into 27,600 gene families by OrthoMCL [35] pipeline, within which 495 gene families were safflower-specific. Compared to olive tree and sesame, safflower shared more gene families with sunflower (Fig. 2b), indicating a closer relationship between safflower and sunflower.

Furthermore, gene family size changes was evaluated by CAFE software [36]. As for safflower, 516 gene families demonstrated under size expansion, while 2,126 gene families indicated under size contraction. Kyoto Encyclopedia of Genes and Genomes (KEGG) pathway enrichment was implemented to the expanded gene families. From which, 225 of 2,751 genes were markedly enriched in fatty acids biosynthesis and metabolism pathways, including linoleic acid metabolism (map00591), alpha-linolenic acid metabolism (map00592) and biosynthesis of unsaturated fatty acids (map01040) (Figure S4). Expansion of gene families involved in fatty acid metabolism especially in unsaturated fatty acids biosynthesis may result in high oil production in safflower.

### WGD in safflower genome

**Fig. 3:**
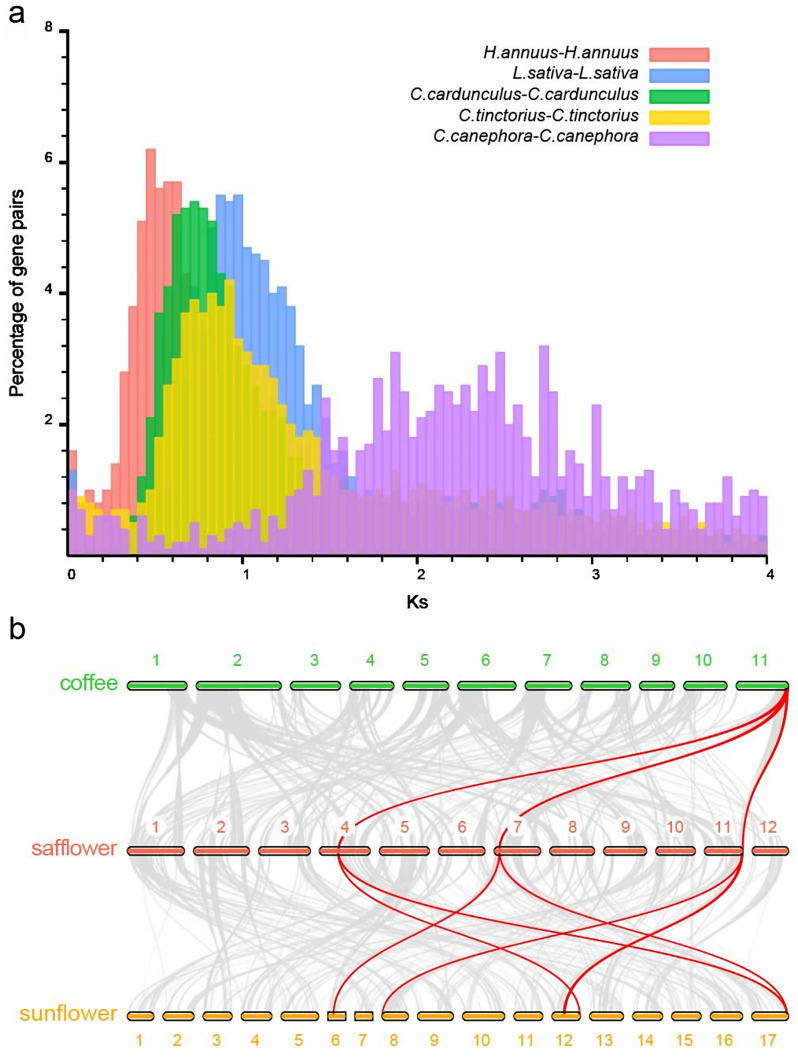
WGD events in *Carthamus tinctorius* evolutionary history. (**a**) Histogram of five species paralog Ks distributions. (**b**) Macrosynteny of gene regions among coffee, safflower and sunflower. Grey lines indicate the synteny blocks between each two species. Red lines highlight the 1:3 between coffee and safflower and 1:2 between safflower and sunflower synteny block corresponding relations

As a member of Compositae family, safflower had a recent whole genome duplication (WGD) event in evolution history (38–50 Mya) (Figure S5). We used WGD pipeline [37] to calculate Ks distribution of paralogs in safflower, sunflower, lettuce, artichoke and coffee tree, respectively (Fig. 3a). After the γ duplication event in eudicots (peak of coffee tree), safflower had experienced another WGD event, which was also found in artichoke and lettuce (Ks ∼ 0.75-1). Besides, this round of duplication was a triplication event, illustrated by the residues of triplication (Fig. 1) and triplicate synteny blocks between coffee tree and safflower. In Compositae, two rounds of WGD events occurred in *Heliantheae* species, which revealed that several 1:2 synteny blocks between safflower and sunflower were existed (Fig. 3b).

### Unsaturated fatty acid biosynthesis pathway

**Fig. 4:**
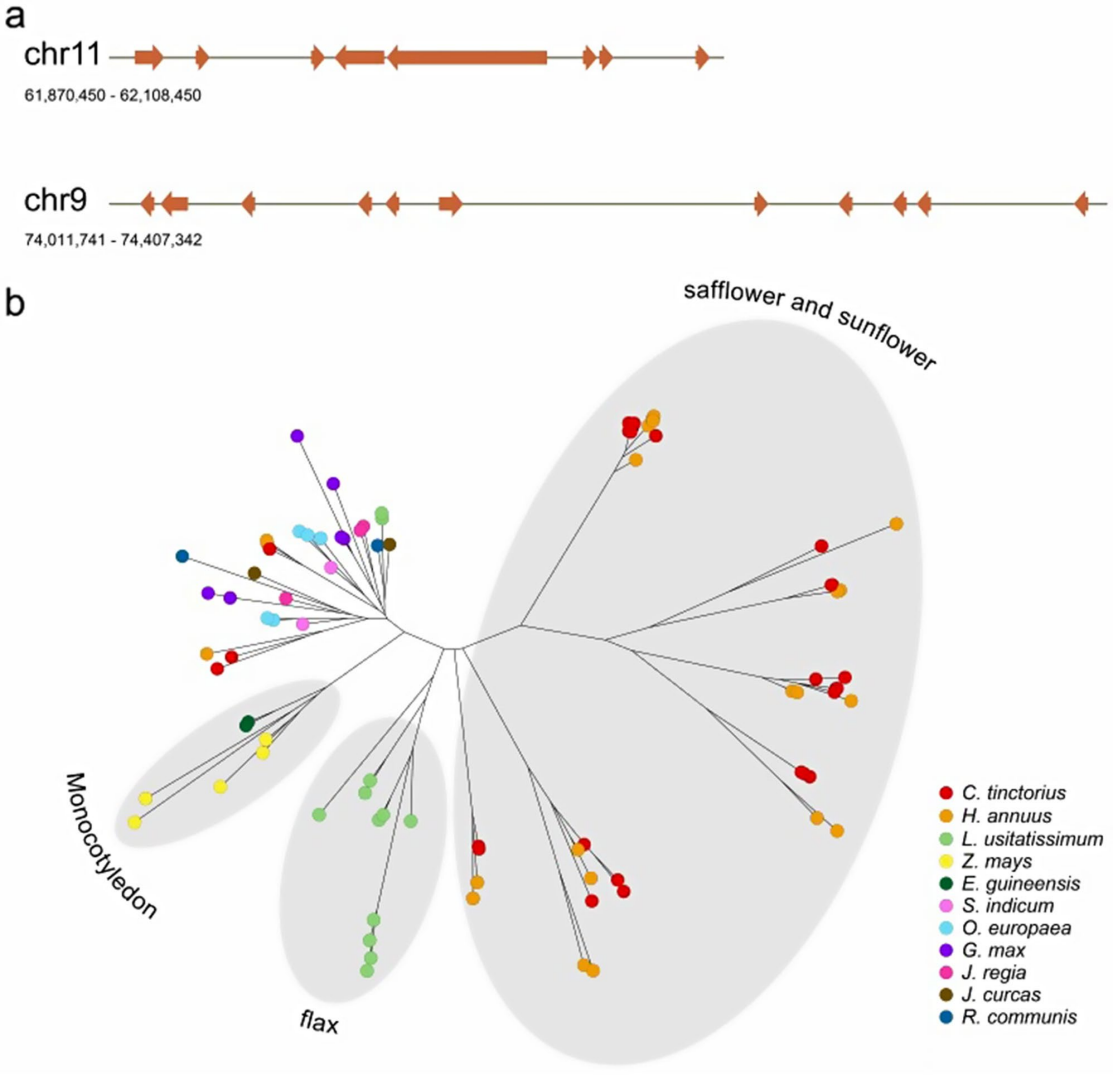
*FAD2* gene clusters. (**a**) Two *FAD2* gene clusters on chromosome 9 and chromosome 11. The line indicates chromosome segment. Brown arrows indicate *FAD2* genes. (**b**) Phylogenetic analysis of the *FAD2* gene family among 11 species [31, 38]. Each circle indicates a *FAD2* gene. Different colors represent different species

We annotated a total of 1,586 genes involved in lipid metabolism in safflower genome. Proteins of 96 genes were functionally enriched in biosynthesis of unsaturated fatty acids pathway (Table S2). In plants, fatty acid desaturases (FAD) catalyse the desaturation reactions of fatty acids. Stearoyl-ACP desaturase (SAD) is a soluble FAD in plastids, transforming stearic acid (C18:0) to oleic acid (C18:1). FAD2 and FAD6 catalyse further desaturation from oleic acid to linoleic acid (C18:2). FAD2 is localised in the endoplasmic reticulum (ER) while FAD6 in the plastid’s inner envelope. In a previous study, researchers have demonstrated the isolation of 11 members of FAD2 family in safflower [6]. In our assembly, 29 copies of *FAD2* genes were annotated, as well as 4 *SAD* genes and 1 *FAD6* gene (Table S3). The *FAD2* genes were amplified by tandem duplication and formed 2 gene clusters located on chromosome 9 and chromosome 11 respectively (Fig. 4a). Phylogenetic analysis of *FAD2* gene family in safflower and other oil crops indicated that *FAD2* gene family were significantly expanded in safflower and sunflower. Also, multi-copies of *FAD2* genes were found in flax genome [38] (Fig. 4b).

We also sequenced transcriptome of seed tissue at five development stages after flowering (days after flowering, DAF) (DAF6, DAF12, DAF18, DAF24, DAF30). Each stage was selected with three duplications. The expression patterns of 96 genes likely involved in the biosynthesis of unsaturated fatty acids pathway were analysed using Mfuzz package [39] (Table S2). The upregulation of *FAD2* genes observed from DAF18 to DAF24 (Figure S6, clusters 2 and 7) implying that linoleic acid biosynthesis could be regulated around the fifth or sixth day after flowering. Moreover, the expression pattern of some genes was significantly enhanced at DAF6, however, as the developmental stages progresses, the expression was supressed (Figure S6, cluster 9). This includes genes related to acyl-CoA oxidase and very-long-chain enoyl-CoA reductase.

### Changes in the fatty acid composition and levels during seed developmental stages

**Fig. 5:**
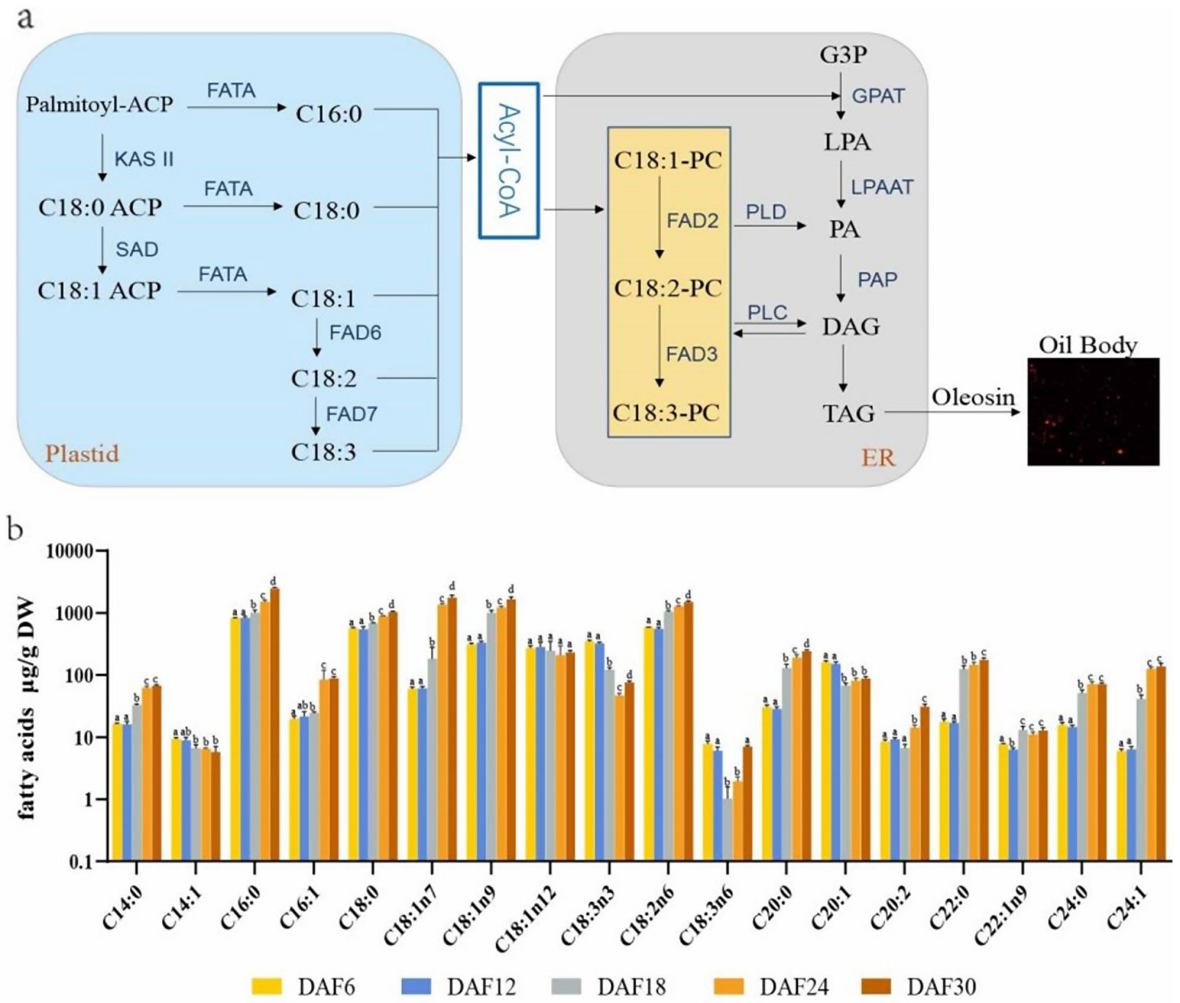
Fatty acids biosynthesis and contents of seed lipids. (**a**) Fatty acid and oil biosynthesis in safflower. KAS II, β-ketoacyl-ACP synthetase; SAD, stromal stearoyl-ACP desaturase; FATA, acyl-ACP thioesterase; G3P, glycerol-3-phosphate; GPAT, glycerol-3-phosphate acyltransferase; LPA, lysophosphatidic acid; LPAAT, Lysophosphatidic acid acyltransferase; PA, phosphatidic acid; FAD2/3/6/7, Fatty acid desaturase; DAG, diglycerides; TAG, triglycerides; (**b**) Fatty acids contents of seed storage lipids in different developing stages

Fatty acids are essential for plant growth and development. FAs are synthesized in plastids and to a large extent transported to the endoplasmic reticulum for modification and lipid assembly. Many genes participate in lipid metabolism within the plastid and endoplasmic reticulum, particularly in fatty acid elongation and desaturation (Fig. 5a). Fatty acid composition and contents are the most important indicators to measure the lipid quality. We examined the fatty acid composition of seed storage lipids in developing seeds. The contents of fatty acids of seeds in five developmental stages was measured by GC-MS. Compositional analyses of seed oil revealed that palmitic acid (C16:0), stearic acid (C18:0), oleic acid (C18:1) and linoleic acid (C18:2) accounted for a predominant proportion of the lipid content in safflower (Fig. 5b). The contents of oleic acid (C18:1) and linoleic acid (C18:2) were found the highest at maturity stage. For instance, C18:1n7, C18:1n9 and C18:2n6 reached 1,776, 1,666, 1,510 µg/g DW, increased by 29.9, 5.4, and 2.6 times when compared to the early stages of grain formation, respectively. According to the data results, C18:3n3 and C18:3n6 did not belong to the high content of PUFA, and their contents decreased first and then increased in the seed developmental stage. In addition to this, most of the fatty acids were increased in the seeds except C14:1, which was decreased. The composition and content of fatty acids in safflower seeds during seed development indicated that the synthesis and accumulation of polyunsaturated fatty acids C18:2 was the main factor determining the oil quality of safflower seeds.

**Fig. 6:**
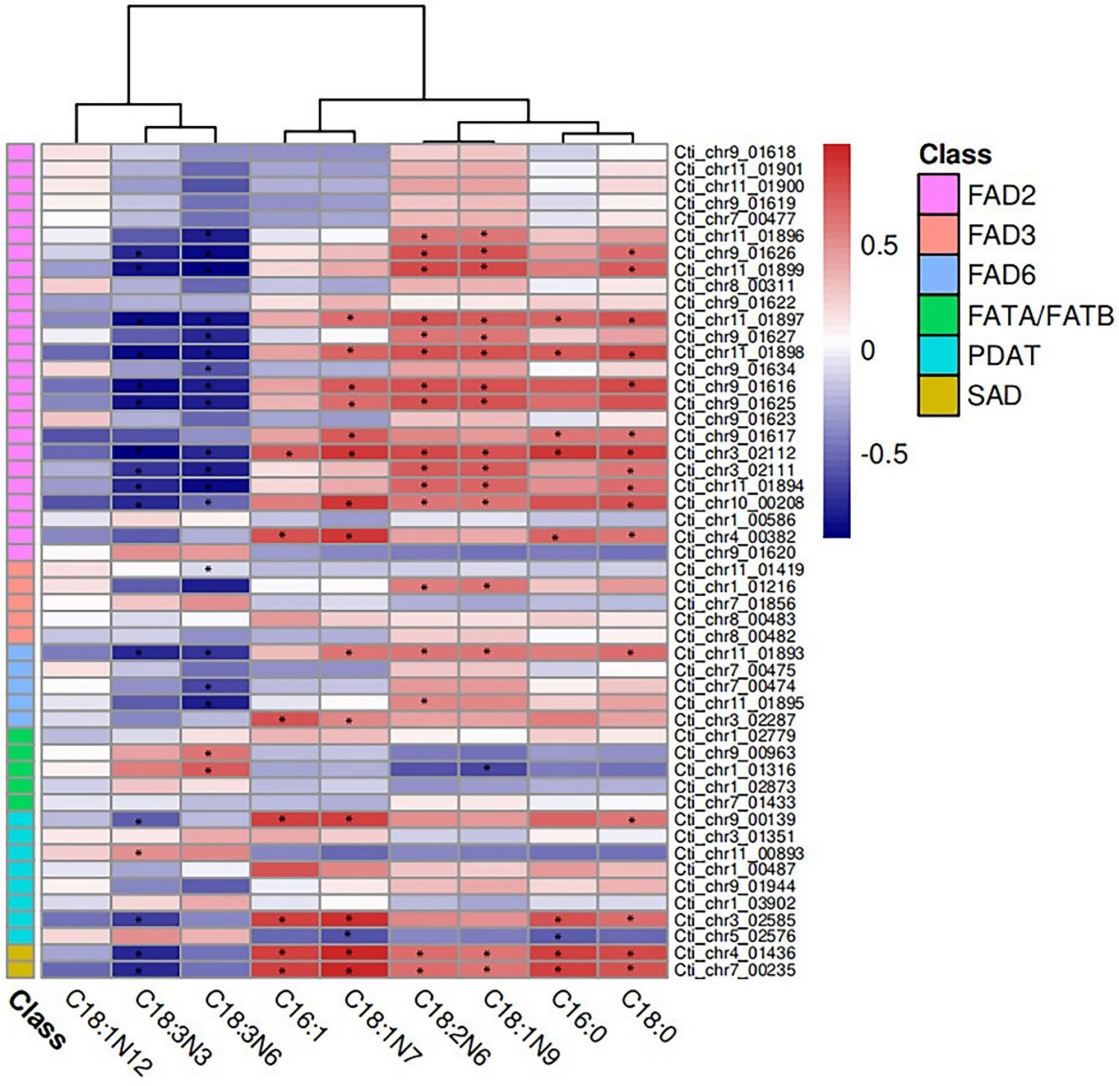
Correlation coefficient between gene expression level and contents of fatty acids. * *p* < 0.05

To gain a better understanding of the relationship between genes and fatty acids species, the Pearson correlation test was performed for the intensity of fatty acids and the expression pattern of genes during the safflower seeds development stage. Our results showed that a total of 28 genes were significantly correlated with C18:1, C18:2 and C18:3 molecular species metabolites that exhibit a Pearson correlation coefficient > 0.7 and p-value < 0.05. Among them, expression pattern of 15 *FAD2* genes (Cti _chr11_01896, Cti_chr9_01626, Cti_chr11_01899, Cti_chr11_01897, Cti_ chr9_01627, Cti_chr11_01898, Cti_chr9_01634, Cti_chr9_01616, Cti_chr9_01625, Cti_chr9_01617, Cti_chr3_02112, Cti_chr3_02111, Cti_chr11_01894, Cti_chr10_00208, Cti_chr4_00382) and 4 *FAD6* genes (Cti_chr11_01893, Cti_chr7_00474, Cti_chr11_01895, Cti_chr3_02287) were positively correlated with C18:1n7, C18:1n9 and C18:2n6 composition patterns during seed developing stages (Fig. 6). Importantly, two *FAD6* genes Cti_chr11_01893 and Cti_chr11_01895 expression patterns showed significant correlation with C18:2 contents during seed development stage. These results indicated that *FAD2* and *FAD6* genes appear to be responsible for the high proportion of C18:2 in developing safflower seeds.

## Discussion

In the present study, we report the complete genome sequence of an economically important crop safflower. We presented valuable insights into the genetic organization of safflower, which facilitates the identification of key functional genes implicated in fatty acid synthesis. These valuable genomic resources could be easily accessible to researchers in the field for future functional and molecular breeding studies. Previous studies on the Compositae have reported genome sequences for *H. annuus* [31], lettuce (*Lactuca sativa*) [32], and globe artichoke (*Cynara cardunculus* var. scolymus) [30]. Those studies have provided scientific resources for comprehensive analyses of genome evolution, functional gene exploration, metabolic pathway construction, and molecular breeding programs.

In light of our results, we revealed a high-quality safflower genome, with a size of 1,061.1 Mb and 12 pseudochromosomes. There are many karyotypes in the ancestor species of safflower with 10, 11, 12, 22, and 32 pairs of chromosomes, many of which are self-incompatibility species [40]. The current karyotype of cultivated safflower could have originated from wild ancestor *C. tinctorius*, with 2n = 24 chromosomes karyotype. This high-quality genome information will be useful for analysing sequences of homologous species, and provides genetic evidence for the nutritional compounds encoded in the safflower genome. In addition, our analysis also revealed that 63.4% of the assembled genome comprises repetitive sequences. This percentage is notably close to that of artichoke (58.4%), yet lower than observed in sunflower (74.7%) and lettuce (74.2%). The relatively larger genome size of sunflower and lettuce could be related to the larger amount of repetitive sequences. It is widely believed that transposable elements play a dominant role in the growth of genome size, and much of the variation in plant genome size can be attributed to the continuous accumulation of these transposable elements. For instance, the sunflower genome is 3.6 Gb and the lettuce genome are 2.38 Gb, which are nearly two to three times of artichoke genome (1.08 Gb) and safflower genome (1.06 Gb). Although, for sunflower genome, the influence of WGD event should be take into consideration regarding the large genome size, our analysis also suggests that repetitive sequences have contributed significantly to the genome size of both sunflower and lettuce. The disparities in repetitive sequence content provide crucial insights into the genomic architecture of these plant species. The higher proportion of repetitive elements in sunflower and lettuce genomes may contribute to their larger genome sizes compared to artichoke and safflower.

First safflower (*Carthamus tinctorius* L.) cultivar ‘Anhui-1’ genome was sequencing using PacBio Sequel (Pacific Biosciences) combined with Illumina Hiseq 2500 sequencing platform and focused on biosynthetic pathways of LA and HSYA [25]. Here, in this work, we sequenced and denovo assembled genome of safflower cultivar ‘Jihong01’ using BGI-SEQ500 and Oxford Nanopore Technologies (ONT) platform and gained another high-quality genome assembly results. Meanwhile, we paid more attention to UFA contents than saturated fatty acid during seed development stages. Seed oil fatty acids composition continues to be important trait for safflower breeding [41]. Recent studies on the molecular mechanisms of lipid metabolism have identified a number of genes that form the genetic basis of this trait. The factors determining seed oil composition were found to be complex. In cultivated safflower, lipid metabolic pathways underlie the natural trait of seed oil content, and candidate genes in the genome were identified to be involved in lipid metabolism and reported before. The unsaturated fatty acid synthesis in ER is important during safflower seed development. Our analyses indicated that the genes involved in fatty acids composition in safflower seeds have undergone expansion during evolution. These analyses may provide essential clues about the biochemical relevance of lipid composition in seeds.

In terms of the fatty acid composition of oil, there is a lower proportion of LA in sesame (C18:2, 32.95–52.94%) [42] than in safflower (C18:2, 63.9–76.1%) [41, 43, 44]. However, the number of genes involved in fatty acid elongation, biosynthesis, and degradation are similar among the genomes of safflower, sunflower, sesame [42], grape [45], capsicum [46], and *Arabidopsis* [47]. A few key enzymes in the desaturation metabolic pathway regulate unsaturated fatty acid biosynthesis in the ER. Genetic evolution, including genome duplication or gene family expansion, is crucial for generating new gene functions and/or for intensifying pathways [48, 49]. Divergence from an ancestral genome can result in an evolutionary bias towards the production of specific natural products. The emergence of duplicates can result in gene expansion, contraction, or loss [50]. *FAD2* gene family encoding enzymes that catalyse linoleic acid biosynthesis has expanded via tandem duplication and formed two gene clusters located on chromosome 9 and chromosome 11 respectively. The result indicated that tandem duplication possibly contributed to the expansion of the gene families in safflower. In particular, the *FAD2* and *FAD6* homologs involved in unsaturated fatty acid biosynthesis showed the highest transcript levels at the DAF18 and DAF24 stage of seed development. Previous studies found that their expression patterns were related to the LA content, with high transcript levels during seed development [41, 51]. These findings provide substantial novel insights into the reasons for the high proportion of LA in safflower oil.

The assembled genome sequence of safflower mostly consisted of repeats, coding and non-coding RNAs, and other related sequences. This information allowed us to reconstruct the evolutionary history of safflower, which includes a large-scale safflower-specific whole-genome duplication events. Candidate genes, including *FAD2* and *FAD6*, which encode key functional enzymes related to LA composition. *FAD2* families were expanded in safflower, and correlation analysis of gene expression alongside contents of fatty acids indicated that the specific *FAD2* and *FAD6* genes could be responsible for the synthesis of a wide range of LA.

## Conclusions

The safflower genome assembly represents a cornerstone for future research programs aimed at exploiting the economic properties of safflower, while also considering agricultural constraints and human nutritional needs and for advancing molecular breeding programs aimed at producing new safflower cultivars. The candidate *FAD2* and *FAD6* genes revealed by our integrated approach provide a genetic resources of unsaturated fatty acid biosynthesis and provide a genetic landscape for safflower germplasm utilization.

### Experimental procedures

#### DNA extracting and sequencing

The plant material of safflower (*Jihong01* cultivar, deposited in Engineering Research Center of Bioreactor and Pharmaceutical Development, Ministry of Education, JLAU) used in this study was identified by Yuanyuan Dong. Safflower variety seedlings (*Jihong01*) collected and planted in an experimental field of Jilin Agriculture University, Changchun City, China were used in this research and stored in Engineering Research Center of Bioreactor and Pharmaceutical Development, Ministry of Education. High-quality genomic DNA from *Jihong01* leaves was extracted using the MolPure® Plant DNA Kit (Yeasen, China). Subsequently, the extraction process focused on selecting large-size fragments, which were accomplished through automated Blue Pippin system. Following this, the DNA underwent treatment involving the end-repair/dA tailing module, and subsequently, it was ligated to an adaptor using the ONT 1D ligation sequencing kit. The prepared library was loaded onto flow cells and subjected to sequencing using the Nanopore PromethION platform.

### Genome assembly

K-mer frequency was calculated by Jellyfish v2.26 [52] and the genome size was estimated using GenomeScope [53]. The initial contigs were assembled by NECAT with default parameters using Nanopore reads longer than 5 kb. The initial assembly was error-corrected by Pilon with short reads. Size of the initial assembly was a little larger than estimated, so we used HaploMerger2 software [27] to remove redundant contigs in the initial assembly. Then, the assembly was error-corrected again. Reads generated by Hi-C library were filtered strictly by HiC-Pro pipeline [54] to remove invalid reads pairs. We used Juicer [55] and 3D *de novo* assembly (3D-DNA) pipeline [56] to anchor contigs to pseudochromosomes.

### Repeat annotation and LTR insertion time

Repetitive sequences were annotated using a combination of *de novo* and homology strategy. We used RepeatModeler [57], LTR_FINDER [58] and TRF [59] software for *de novo* repeats identification based on repetitive sequences features. Then RepeatMasker and RepeatProteinMask were used to annotate transposon elements based on RepBase. LTR insertion time was estimated based on the divergence of LTR pairs. Intact LTRs were identified using LTR_FINDER software in the four Compositae genomes. Then we used MUSCLE [60] to align LTR pairs and distmat to calculate *K* values under the Kimura two-parameter model. With the *K* values of LTR pairs, the insertion time was calculated using formula *T = K/2r*, where *r* was the rate of nucleotide substitution and set as 7 × 10^− 9^ per site per generation here [61, 62].

### Genes prediction and function annotation

Protein-coding genes were predicted based on both homolog proteins and transcripts. Proteins of seven plants species (*Arachis hypogaea* (GCF_003086295.2), *Brassica napus* (GCF_000686985.2), *Glycine max* (GCF_000004515.5), *Helianthus annuus* (GCF_002127325.1), *Lactuca sativa* (GCF_002870075.1), *Ricinus communis* (GCF_000151685.1) and *Sesamum indicum* (GCF_000512975.1)) was downloaded from NCBI database. We first aligned these proteins with the assembly using BLAT [63], then the alignment was input to Genewise [64] to get homology annotations. RNA-seq reads were mapped to the assembly using HISAT2 [65] and transcripts were assembled using StringTie [66]. All of the evidences were integrated to the final protein-coding gene set by GLEAN [67]. Protein-coding genes were assessed for conserved protein domains in the ProDom, ProSiteProfiles, SMART, PANTHER, Pfam, PIRSF and ProSitePatterns databases using InterProScan [68]. Also, amino acid sequences were aligned to the following protein databases: Swiss-Prot, TrEMBL, Kyoto Encyclopaedia of Genes and Genomes (KEGG) using BLASTP (e-value < 1e-5) for function annotation.

### Comparative analysis

All protein-coding gene sequences of the 10 oil plant species (*Elaeis guineensis* (GCF_000442705.1), *Glycine max*, *Helianthus annuus*, *Jatropha curcas* (GCF_000696525.1), *Juglans regia* (GCF_001411555.1), *Linum usitatissimum* (Linum usitatissimum v1.0), *Olea europaea* (GCF_002742605.1), *Ricinus communis*, *Sesamum indicum* and *Zea mays* (GCF_000005005.2)) were downloaded from the NCBI or Phytozome database. The longest transcript of each gene without frame shift or internal termination was selected and translated into amino acid sequences for subsequent analyses. First, we used BLASTP for an all-to-all proteins alignment under the e-value of 1e-5. Then the ortholog genes were clustered into groups using OrthoMCL with a Markov inflation index of 1.5 and a maximum e-value of 1e-5. One-to-one single-copy ortholog groups were joined to a super-gene (single-copy orthologous genes are composed of head-to-tail connections) and aligned using MUSCLE [60]. Phylogenetic tree was constructed using RAxML [69] with *Z. mays* and *E. guineensis* as outgroups, under GTR + Optimization of substitution rates and GAMMA model of rate heterogeneity. The divergence time was estimated using MCMCTREE in PAML packages [70] based on HKY85 model and correlated rates molecular clock model. The size changes of each gene families were calculated by CAFE [36] with the random birth and death model.

### WGD events and synteny

We used WGD software [37] to identify WGD events in the evolutionary process of safflower, sunflower, lettuce, artichoke (GCF_002870075.1) and coffee tree (AUK_PRJEB4211_v1). Synteny blocks between safflower and sunflower and between safflower and coffee tree were identified and displayed using jcvi packages [71].

### Identification of unsaturated fatty acids biosynthesis genes

We used BLASTP to identify safflower FAD2 and FAD6 based on amino acids sequences of *Arabidopsis thaliana* FAD2 (NP_187819.1), FAD6 (NP_194824.1) with e-value of 1e-10.

### Fatty acids content analysis

Safflower seeds (*Jihong01)* were sourced from Engineering Research Center of Bioreactor and Pharmaceutical Development, Ministry of Education, JLAU. Dry seed samples (DAF6 (6 days after flowering), DAF12, DAF18, DAF24, DAF30) were collected at five different stages of seed development for the purpose of analysing fatty acid content and composition. Methyl esters (FAME) were prepared from each seed sample. Subsequently, quantitative analysis of fatty acids within these FAME samples was conducted utilizing Gas Chromatography-Mass Spectrometry (GC-MS) through the Agilent Technologies 6890 N/5975B system. The methods employed for this analysis were in accordance with those described by Ecker et al. [72]. The fatty acids present, including saturated fatty acids (SFAs), monounsaturated fatty acids (MUFAs), and polyunsaturated fatty acids (PUFAs), were subjected to quantitative calculations. These calculations were carried out based on a standard fatty acid methyl ester mix.

### RNA sequencing

Total RNA was extracted from the 5 different developing seeds (DAF6 (6 days after flowering), DAF12, DAF18, DAF24, DAF30) using a TRIzol Plus RNA Purification Kit following the manufacturer’s instructions. RNA integrity and quantity were confirmed using an Agilent 2100 Bioanalyzer. The mRNA was hybridized with an Oligo(dT) probe and captured using magnetic beads. Subsequently, the mRNA was fragmented at high temperature and reverse-transcribed into first-strand DNA. This first-strand DNA served as a template for the synthesis of second-strand DNA, resulting in the formation of double-stranded DNA (dsDNA). Adaptors with dTTP tails were ligated to both ends of the dsDNA fragments. The ligation products were then amplified via PCR and circularized to generate a single-stranded circular (ssCir) library. The ssCir library was further amplified through rolling circle amplification (RCA) to produce DNA nanoballs (DNB). Finally, the DNBs were loaded onto a flow cell and sequenced using the DNBSEQ platform. Each sample was sequenced three times in triplicate.

### Electronic supplementary material

Below is the link to the electronic supplementary material.

Supplementary Material 1

## Data Availability

The raw sequence reads were deposited in China National GeneBank DataBase (CNGB db) under Project No. CNP0004859 and CNP0004861.
