# Supplementary material for: The Carthamus tinctorius L. genome sequence provides insights into synthesis of unsaturated fatty acids
Source: BMC Genomics. 2024 May 23;25:510. doi: 10.1186/s12864-024-10405-z (PMC11112859; doi:10.1186/s12864-024-10405-z)
Supplement: Supplementary file 1 — Supplementary Material 1 [file 12864_2024_10405_MOESM1_ESM.docx]

**Title:** The *Carthamus tinctorius L.* genome sequence provides insights into synthesis of unsaturated fatty acids

Authors: Yuanyuan Dong^1, #^, Xiaojie Wang^2, #^, Naveed Ahmad^1^, Yepeng Sun^1^, Yuanxin Wang^1^, Xiuming Liu^1^, Na Yao^1^, Yang Jing^1^, Linna Du^1^, Xiaowei Li^1^, Nan Wang^1^, Weican Liu^1^, Fawei Wang^1^, Xiaokun Li^2^, Haiyan Li^3,*^

^1^ Engineering Research Center of Bioreactor and Pharmaceutical Development, Ministry of Education, College of Life Sciences, Jilin Agricultural University, Changchun 130118, China.

^2^ School of Pharmaceutical Science, Key Laboratory of Biotechnology and Pharmaceutical Engineering of Zhejiang Province, Wenzhou Medical University, Wenzhou 325035, China.

^3^ College of Tropical Crops, Hainan University, Haikou 570228, China.

^#^ These authors contributed equally to this work.

* **Corresponding authors:**

* Haiyan Li, hyli99@163.com

**Authors’ email addresses:**

Yuanyuan Dong, yuanyuand@jlau.edu.cn;

Xiaojie Wang, 18858811123@126.com;

Naveed Ahmad, naveedjlau@gmail.com

Yepeng Sun, 404760896@qq.com

YuanXin Wang, 2755276161@qq.com;

Xiumin Liu, xiuming1211@163.com;

Na Yao, yaona801103@aliyun.com;

Jing Yang, yangjing5122010@163.com;

Linna Du, dulinna0918@163.com;

Xiaowei Li, xiaoweili1206@163.com;

Nan Wang, wangnanlunwen@126.com;

Weican Liu, [liuweican602@163.com](mailto:liuweican602@163.com);

Fawei Wang, [fw-1980@163.com](mailto:fw-1980@163.com);

Xiaokun Li, xiaokunli@163.net;

Haiyan Li, hyli99@163.com.


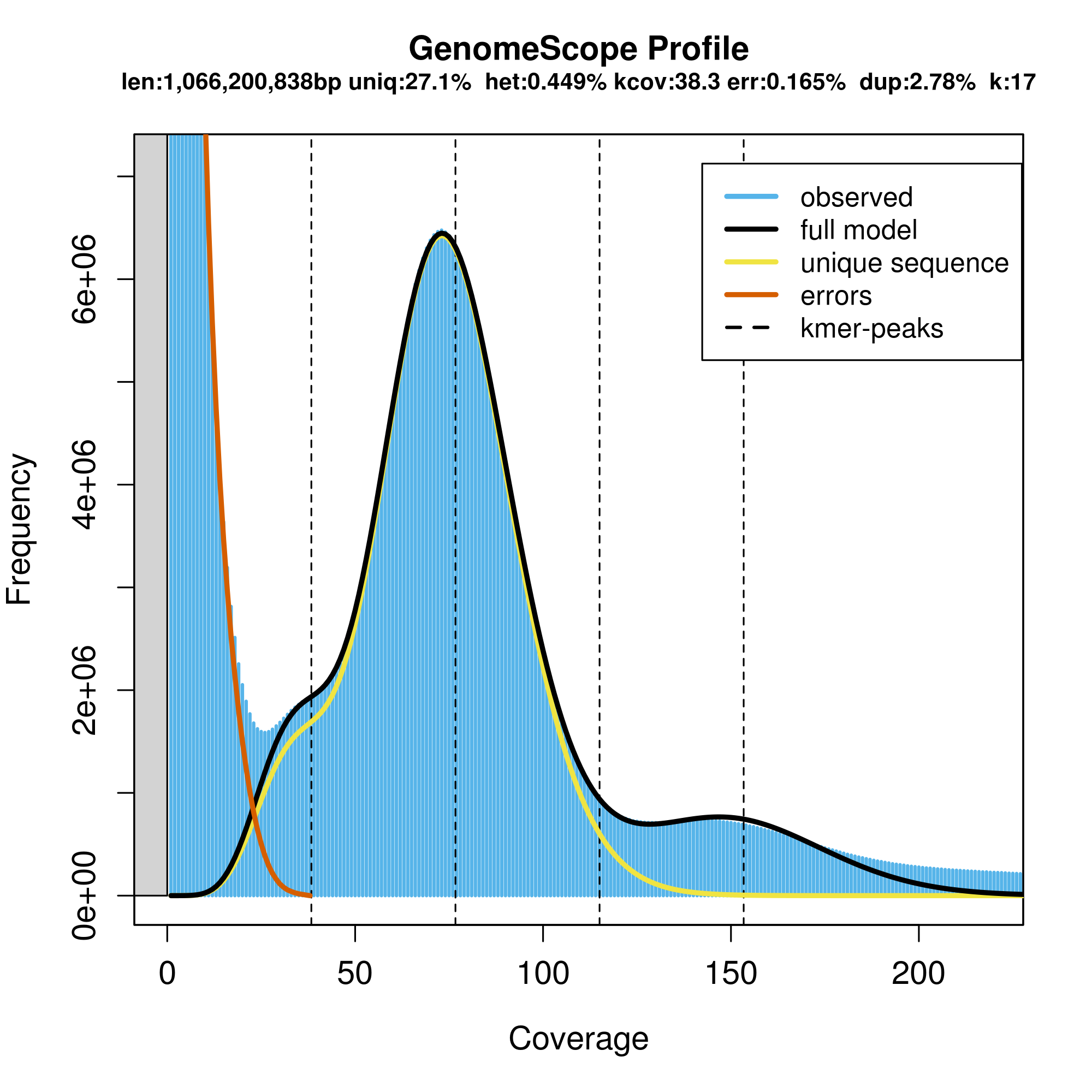


**Figure S1**. 17-mer profile plot of the *Carthamus tinctorius* L. pair-end library


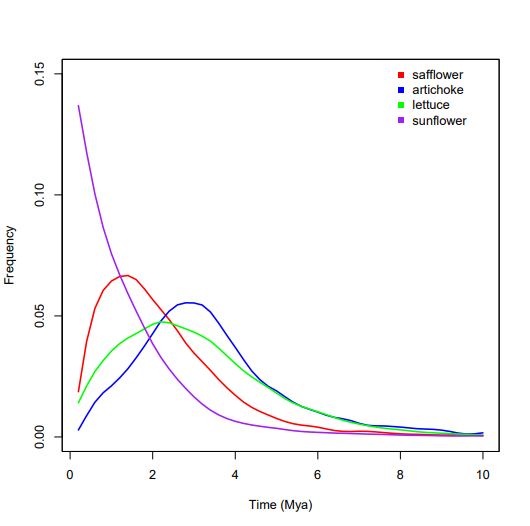


**Figure S2**. Estimate of LTR insertion time


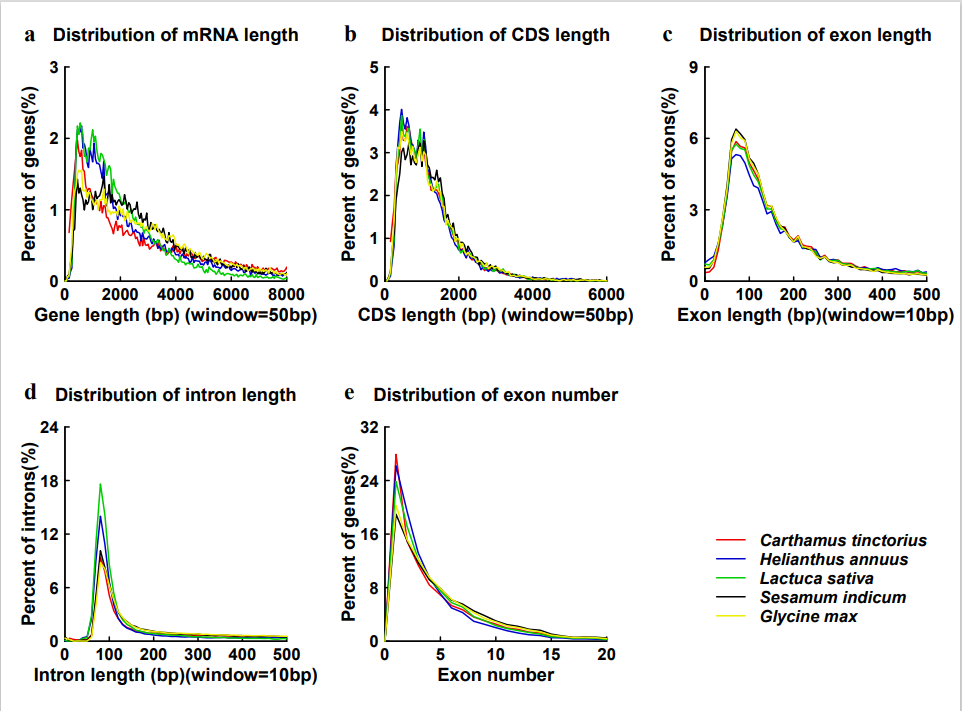
\

**Figure S3**. Distribution of (a) mRNA length, (b) CDS length, (c) exon length, (d) intron length and (e) exon number of protein coding genes in safflower and related species.


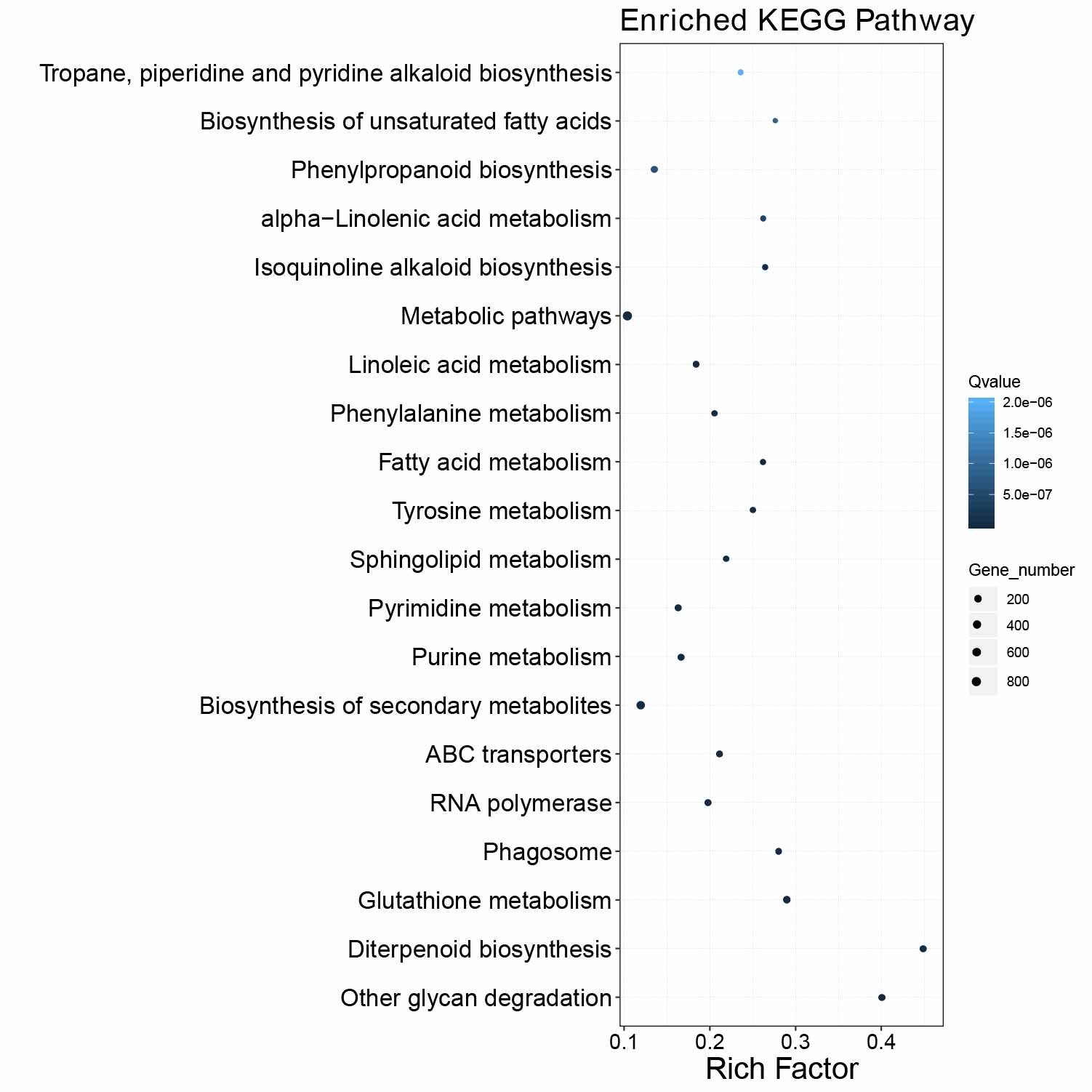


**Figure S4**. KEGG pathway enrichment of significant expanded gene families


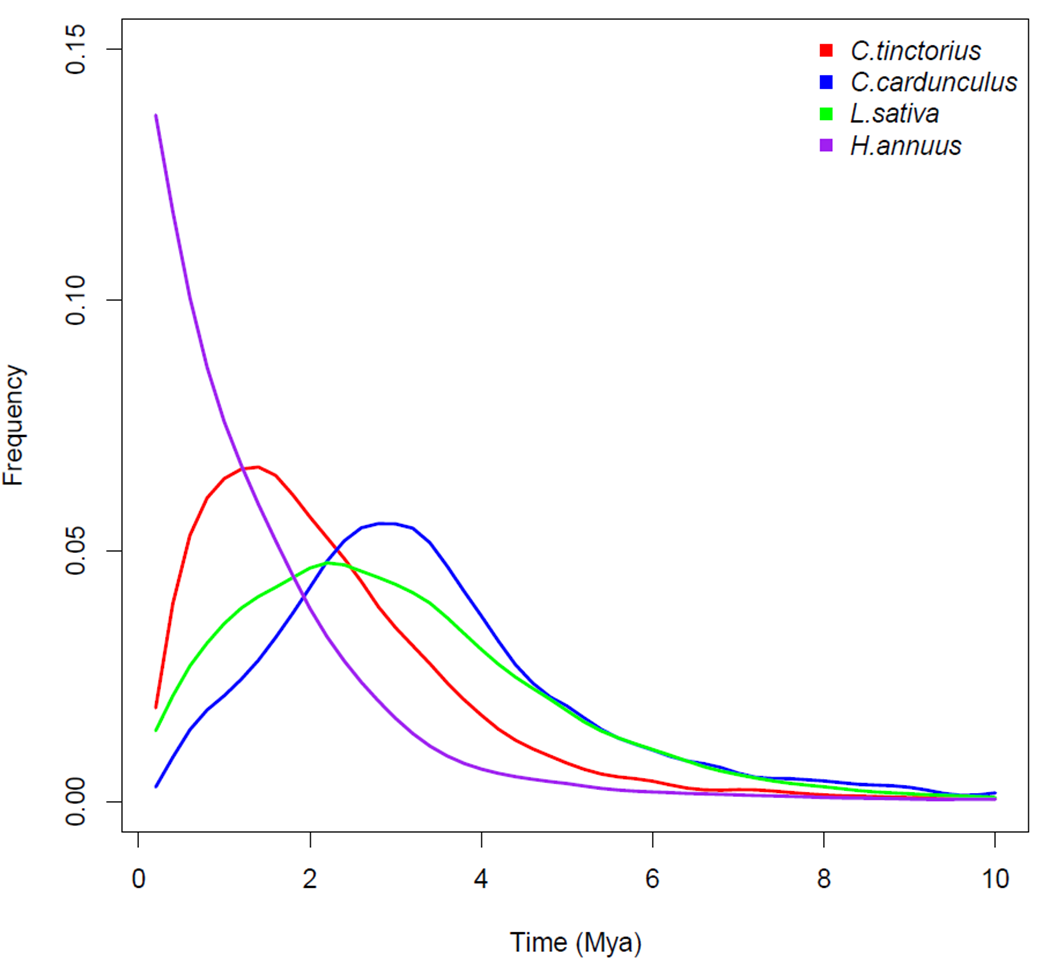


**Figure S5.** Whole genome duplication (WGD) event analysis of safflower


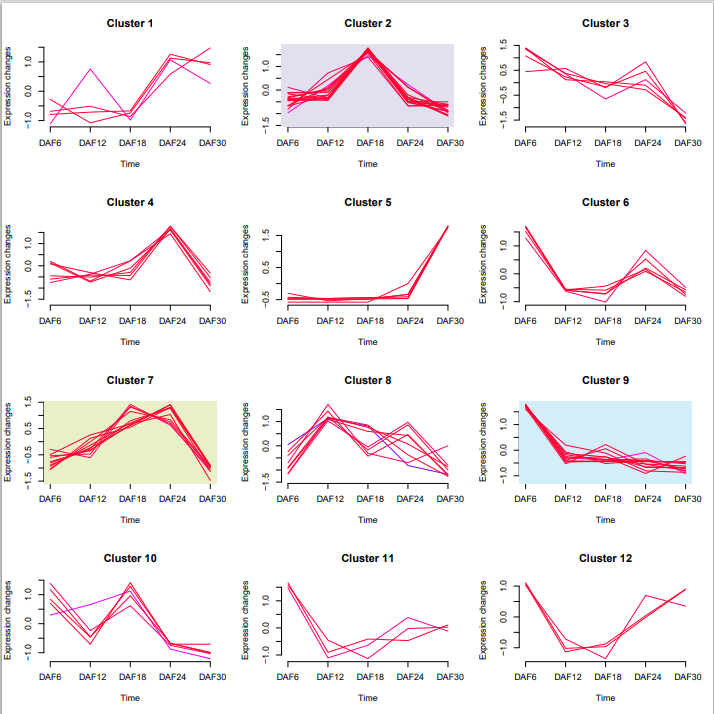


**Figure S6**. Expression patterns of safflower unsaturated fatty acids biosynthesis genes during seed development

**Table S1.** Statistics of safflower genome assembly and annotation

| Assembly size (Mb) | 1061.1 |
| --- | --- |
| Number of contigs | 452 |
| Contig N50 (Mb) | 8.8 |
| Number of scaffolds | 114 |
| Scaffold N50 (Mb) | 87.1 |
| Number of pseudochromosomes | 12 |
| Percent anchored to pseudochromosomes (%) | 98.82 |
| GC content (%) | 38.37 |
| Complete BUSCOs (%) | 90.3 |
| Fragment BUSCOs (%) | 2.4 |
| Missing BUSCOs (%) | 7.3 |
| Repeat sequences (%) | 63.4% |
| Protein coding genes | 32,379 |

**Table S2.** Genes in biosynthesis of unsaturated fatty acids pathway

| Gene_ID | ko | definition |
| --- | --- | --- |
| Cti_chr10_00912 | K00059 | 3-oxoacyl-[acyl-carrier protein] reductase [EC:1.1.1.100] |
| Cti_chr3_02146 | K00059 | 3-oxoacyl-[acyl-carrier protein] reductase [EC:1.1.1.100] |
| Cti_chr4_00272 | K00059 | 3-oxoacyl-[acyl-carrier protein] reductase [EC:1.1.1.100] |
| Cti_chr7_00820 | K00059 | 3-oxoacyl-[acyl-carrier protein] reductase [EC:1.1.1.100] |
| Cti_chr8_00170 | K00059 | 3-oxoacyl-[acyl-carrier protein] reductase [EC:1.1.1.100] |
| Cti_chr8_00199 | K00059 | 3-oxoacyl-[acyl-carrier protein] reductase [EC:1.1.1.100] |
| Cti_chr9_01668 | K00059 | 3-oxoacyl-[acyl-carrier protein] reductase [EC:1.1.1.100] |
| Cti_chr9_01669 | K00059 | 3-oxoacyl-[acyl-carrier protein] reductase [EC:1.1.1.100] |
| Cti_chr9_01670 | K00059 | 3-oxoacyl-[acyl-carrier protein] reductase [EC:1.1.1.100] |
| Cti_chr9_01671 | K00059 | 3-oxoacyl-[acyl-carrier protein] reductase [EC:1.1.1.100] |
| Cti_scaffold74_00139 | K00059 | 3-oxoacyl-[acyl-carrier protein] reductase [EC:1.1.1.100] |
| Cti_scaffold74_00214 | K00059 | 3-oxoacyl-[acyl-carrier protein] reductase [EC:1.1.1.100] |
| Cti_scaffold74_00367 | K00059 | 3-oxoacyl-[acyl-carrier protein] reductase [EC:1.1.1.100] |
| Cti_chr10_00706 | K00232 | acyl-CoA oxidase [EC:1.3.3.6] |
| Cti_chr10_01035 | K00232 | acyl-CoA oxidase [EC:1.3.3.6] |
| Cti_chr10_01515 | K00232 | acyl-CoA oxidase [EC:1.3.3.6] |
| Cti_chr10_01517 | K00232 | acyl-CoA oxidase [EC:1.3.3.6] |
| Cti_chr1_00646 | K00232 | acyl-CoA oxidase [EC:1.3.3.6] |
| Cti_chr11_00263 | K00232 | acyl-CoA oxidase [EC:1.3.3.6] |
| Cti_chr2_00135 | K00232 | acyl-CoA oxidase [EC:1.3.3.6] |
| Cti_chr2_01685 | K00232 | acyl-CoA oxidase [EC:1.3.3.6] |
| Cti_chr2_02588 | K00232 | acyl-CoA oxidase [EC:1.3.3.6] |
| Cti_chr3_00737 | K00232 | acyl-CoA oxidase [EC:1.3.3.6] |
| Cti_chr3_01554 | K00232 | acyl-CoA oxidase [EC:1.3.3.6] |
| Cti_chr3_02109 | K00232 | acyl-CoA oxidase [EC:1.3.3.6] |
| Cti_chr4_01345 | K00232 | acyl-CoA oxidase [EC:1.3.3.6] |
| Cti_chr4_01347 | K00232 | acyl-CoA oxidase [EC:1.3.3.6] |
| Cti_chr5_00227 | K00232 | acyl-CoA oxidase [EC:1.3.3.6] |
| Cti_chr7_00010 | K00232 | acyl-CoA oxidase [EC:1.3.3.6] |
| Cti_chr8_00111 | K00232 | acyl-CoA oxidase [EC:1.3.3.6] |
| Cti_chr8_00256 | K00232 | acyl-CoA oxidase [EC:1.3.3.6] |
| Cti_chr8_00487 | K00232 | acyl-CoA oxidase [EC:1.3.3.6] |
| Cti_chr8_00775 | K00232 | acyl-CoA oxidase [EC:1.3.3.6] |
| Cti_chr9_00572 | K00232 | acyl-CoA oxidase [EC:1.3.3.6] |
| Cti_chr9_00871 | K00232 | acyl-CoA oxidase [EC:1.3.3.6] |
| Cti_chr9_01446 | K00232 | acyl-CoA oxidase [EC:1.3.3.6] |
| Cti_chr9_02046 | K00232 | acyl-CoA oxidase [EC:1.3.3.6] |
| Cti_chr7_01048 | K01062 | platelet-activating factor acetylhydrolase [EC:3.1.1.47] |
| Cti_chr5_02083 | K01068 | acyl-coenzyme A thioesterase 1/2/4 [EC:3.1.2.2] |
| Cti_chr5_02084 | K01068 | acyl-coenzyme A thioesterase 1/2/4 [EC:3.1.2.2] |
| Cti_chr1_03864 | K03921 | acyl-[acyl-carrier-protein] desaturase [EC:1.14.19.2 1.14.19.11 1.14.19.26] |
| Cti_chr2_02009 | K03921 | acyl-[acyl-carrier-protein] desaturase [EC:1.14.19.2 1.14.19.11 1.14.19.26] |
| Cti_chr2_02013 | K03921 | acyl-[acyl-carrier-protein] desaturase [EC:1.14.19.2 1.14.19.11 1.14.19.26] |
| Cti_chr5_00769 | K03921 | acyl-[acyl-carrier-protein] desaturase [EC:1.14.19.2 1.14.19.11 1.14.19.26] |
| Cti_chr10_00049 | K07513 | acetyl-CoA acyltransferase 1 [EC:2.3.1.16] |
| Cti_chr11_01169 | K07513 | acetyl-CoA acyltransferase 1 [EC:2.3.1.16] |
| Cti_chr8_00645 | K07513 | acetyl-CoA acyltransferase 1 [EC:2.3.1.16] |
| Cti_chr10_02151 | K10249 | elongation of very long chain fatty acids protein 4 [EC:2.3.1.199] |
| Cti_chr10_02148 | K10251 | 17beta-estradiol 17-dehydrogenase/very-long-chain 3-oxoacyl-CoA reductase [EC:1.1.1.62 1.1.1.330] |
| Cti_chr4_00901 | K10251 | 17beta-estradiol 17-dehydrogenase/very-long-chain 3-oxoacyl-CoA reductase [EC:1.1.1.62 1.1.1.330] |
| Cti_chr4_00902 | K10251 | 17beta-estradiol 17-dehydrogenase/very-long-chain 3-oxoacyl-CoA reductase [EC:1.1.1.62 1.1.1.330] |
| Cti_chr6_02134 | K10251 | 17beta-estradiol 17-dehydrogenase/very-long-chain 3-oxoacyl-CoA reductase [EC:1.1.1.62 1.1.1.330] |
| Cti_chr10_00208 | K10256 | omega-6 fatty acid desaturase/acyl-lipid omega-6 desaturase (Delta-12 desaturase) [EC:1.14.19.6 1.14.19.22] |
| Cti_chr1_00586 | K10256 | omega-6 fatty acid desaturase/acyl-lipid omega-6 desaturase (Delta-12 desaturase) [EC:1.14.19.6 1.14.19.22] |
| Cti_chr11_01893 | K10256 | omega-6 fatty acid desaturase/acyl-lipid omega-6 desaturase (Delta-12 desaturase) [EC:1.14.19.6 1.14.19.22] |
| Cti_chr11_01894 | K10256 | omega-6 fatty acid desaturase/acyl-lipid omega-6 desaturase (Delta-12 desaturase) [EC:1.14.19.6 1.14.19.22] |
| Cti_chr11_01895 | K10256 | omega-6 fatty acid desaturase/acyl-lipid omega-6 desaturase (Delta-12 desaturase) [EC:1.14.19.6 1.14.19.22] |
| Cti_chr11_01896 | K10256 | omega-6 fatty acid desaturase/acyl-lipid omega-6 desaturase (Delta-12 desaturase) [EC:1.14.19.6 1.14.19.22] |
| Cti_chr11_01897 | K10256 | omega-6 fatty acid desaturase/acyl-lipid omega-6 desaturase (Delta-12 desaturase) [EC:1.14.19.6 1.14.19.22] |
| Cti_chr11_01898 | K10256 | omega-6 fatty acid desaturase/acyl-lipid omega-6 desaturase (Delta-12 desaturase) [EC:1.14.19.6 1.14.19.22] |
| Cti_chr11_01899 | K10256 | omega-6 fatty acid desaturase/acyl-lipid omega-6 desaturase (Delta-12 desaturase) [EC:1.14.19.6 1.14.19.22] |
| Cti_chr11_01900 | K10256 | omega-6 fatty acid desaturase/acyl-lipid omega-6 desaturase (Delta-12 desaturase) [EC:1.14.19.6 1.14.19.22] |
| Cti_chr11_01901 | K10256 | omega-6 fatty acid desaturase/acyl-lipid omega-6 desaturase (Delta-12 desaturase) [EC:1.14.19.6 1.14.19.22] |
| Cti_chr3_02111 | K10256 | omega-6 fatty acid desaturase/acyl-lipid omega-6 desaturase (Delta-12 desaturase) [EC:1.14.19.6 1.14.19.22] |
| Cti_chr3_02112 | K10256 | omega-6 fatty acid desaturase/acyl-lipid omega-6 desaturase (Delta-12 desaturase) [EC:1.14.19.6 1.14.19.22] |
| Cti_chr4_00382 | K10256 | omega-6 fatty acid desaturase/acyl-lipid omega-6 desaturase (Delta-12 desaturase) [EC:1.14.19.6 1.14.19.22] |
| Cti_chr7_00474 | K10256 | omega-6 fatty acid desaturase/acyl-lipid omega-6 desaturase (Delta-12 desaturase) [EC:1.14.19.6 1.14.19.22] |
| Cti_chr7_00475 | K10256 | omega-6 fatty acid desaturase/acyl-lipid omega-6 desaturase (Delta-12 desaturase) [EC:1.14.19.6 1.14.19.22] |
| Cti_chr7_00477 | K10256 | omega-6 fatty acid desaturase/acyl-lipid omega-6 desaturase (Delta-12 desaturase) [EC:1.14.19.6 1.14.19.22] |
| Cti_chr8_00311 | K10256 | omega-6 fatty acid desaturase/acyl-lipid omega-6 desaturase (Delta-12 desaturase) [EC:1.14.19.6 1.14.19.22] |
| Cti_chr9_01616 | K10256 | omega-6 fatty acid desaturase/acyl-lipid omega-6 desaturase (Delta-12 desaturase) [EC:1.14.19.6 1.14.19.22] |
| Cti_chr9_01617 | K10256 | omega-6 fatty acid desaturase/acyl-lipid omega-6 desaturase (Delta-12 desaturase) [EC:1.14.19.6 1.14.19.22] |
| Cti_chr9_01618 | K10256 | omega-6 fatty acid desaturase/acyl-lipid omega-6 desaturase (Delta-12 desaturase) [EC:1.14.19.6 1.14.19.22] |
| Cti_chr9_01619 | K10256 | omega-6 fatty acid desaturase/acyl-lipid omega-6 desaturase (Delta-12 desaturase) [EC:1.14.19.6 1.14.19.22] |
| Cti_chr9_01620 | K10256 | omega-6 fatty acid desaturase/acyl-lipid omega-6 desaturase (Delta-12 desaturase) [EC:1.14.19.6 1.14.19.22] |
| Cti_chr9_01622 | K10256 | omega-6 fatty acid desaturase/acyl-lipid omega-6 desaturase (Delta-12 desaturase) [EC:1.14.19.6 1.14.19.22] |
| Cti_chr9_01623 | K10256 | omega-6 fatty acid desaturase/acyl-lipid omega-6 desaturase (Delta-12 desaturase) [EC:1.14.19.6 1.14.19.22] |
| Cti_chr9_01625 | K10256 | omega-6 fatty acid desaturase/acyl-lipid omega-6 desaturase (Delta-12 desaturase) [EC:1.14.19.6 1.14.19.22] |
| Cti_chr9_01626 | K10256 | omega-6 fatty acid desaturase/acyl-lipid omega-6 desaturase (Delta-12 desaturase) [EC:1.14.19.6 1.14.19.22] |
| Cti_chr9_01627 | K10256 | omega-6 fatty acid desaturase/acyl-lipid omega-6 desaturase (Delta-12 desaturase) [EC:1.14.19.6 1.14.19.22] |
| Cti_chr9_01634 | K10256 | omega-6 fatty acid desaturase/acyl-lipid omega-6 desaturase (Delta-12 desaturase) [EC:1.14.19.6 1.14.19.22] |
| Cti_chr1_00501 | K10258 | very-long-chain enoyl-CoA reductase [EC:1.3.1.93] |
| Cti_chr1_00784 | K10258 | very-long-chain enoyl-CoA reductase [EC:1.3.1.93] |
| Cti_chr1_00785 | K10258 | very-long-chain enoyl-CoA reductase [EC:1.3.1.93] |
| Cti_chr1_01059 | K10258 | very-long-chain enoyl-CoA reductase [EC:1.3.1.93] |
| Cti_chr11_01509 | K10258 | very-long-chain enoyl-CoA reductase [EC:1.3.1.93] |
| Cti_chr11_01732 | K10258 | very-long-chain enoyl-CoA reductase [EC:1.3.1.93] |
| Cti_chr11_01733 | K10258 | very-long-chain enoyl-CoA reductase [EC:1.3.1.93] |
| Cti_chr8_02329 | K10258 | very-long-chain enoyl-CoA reductase [EC:1.3.1.93] |
| Cti_chr8_02330 | K10258 | very-long-chain enoyl-CoA reductase [EC:1.3.1.93] |
| Cti_chr12_01111 | K10703 | very-long-chain (3R)-3-hydroxyacyl-CoA dehydratase [EC:4.2.1.134] |
| Cti_chr9_01554 | K10703 | very-long-chain (3R)-3-hydroxyacyl-CoA dehydratase [EC:4.2.1.134] |
| Cti_chr3_02029 | K11147 | dehydrogenase/reductase SDR family member 4 [EC:1.1.-.-] |
| Cti_chr3_02144 | K11147 | dehydrogenase/reductase SDR family member 4 [EC:1.1.-.-] |
| Cti_chr9_00599 | K11147 | dehydrogenase/reductase SDR family member 4 [EC:1.1.-.-] |
| Cti_chr12_01704 | K18643 | katanin p80 WD40 repeat-containing subunit B1 |

**Table S3.** SAD, FAD6 and FAD2 genes in safflower genome

| Name | Gene_ID | Location |
| --- | --- | --- |
| SAD | Cti_chr5_00769 | Chr5: 11,058,079 - 11,063,551 |
|  | Cti_chr1_03864 | Chr1: 82,519,659 - 82,521,273 |
|  | Cti_chr2_02009 | Chr2: 81,984,115 - 81,985,794 |
|  | Cti_chr2_02013 | Chr2: 82,133,558 - 82,150,351 |
| FAD6 | Cti_Chr5:_02287 | Chr5: 91,437,591 - 91,445,070 |
| FAD2 | Cti_chr4_00382 | Chr4: 24,060,249 - 24,061,391 |
|  | Cti_chr10_00208 | Chr10: 2,477,936 - 2,479,096 |
|  | Cti_chr8_00311 | Chr8: 5,957,912 - 5,959,063 |
|  | Cti_chr1_00586 | Chr1: 8,183,715 - 8,184,839 |
|  | Cti_chr7_00477 | Chr7: 6,397,369 - 6,398,487 |
|  | Cti_chr7_00475 | Chr7: 6,317,521 - 6,318,666 |
|  | Cti_chr7_00474 | Chr7: 6,292,024 - 6,293,166 |
|  | Cti_chr9_01616 | Chr9: 74,011,741 - 74,012,877 |
|  | Cti_chr9_01617 | Chr9: 74,020,084 - 74,027,093 |
|  | Cti_chr9_01618 | Chr9: 74,054,265 - 74,055,401 |
|  | Cti_chr9_01619 | Chr9: 74,103,628 - 74,104,773 |
|  | Cti_chr9_01620 | Chr9: 74,115,239 - 74,116,384 |
|  | Cti_chr9_01622 | Chr9: 74,137,867 - 74,143,623 |
|  | Cti_chr9_01623 | Chr9: 74,271,428 - 74,272,591 |
|  | Cti_chr9_01625 | Chr9: 74,306,640 - 74,307,776 |
|  | Cti_chr9_01626 | Chr9: 74,329,447 - 74,330,583 |
|  | Cti_chr9_01627 | Chr9: 74,339,750 - 74,340,886 |
|  | Cti_chr9_01634 | Chr9: 74,406,179 - 74,407,342 |
|  | Cti_chr11_01901 | Chr11: 62,107,320 - 62,108,450 |
|  | Cti_chr11_01900 | Chr11: 62,065,880 - 62,066,147 |
|  | Cti_chr11_01899 | Chr11: 62,058,959 - 62,060,089 |
|  | Cti_chr11_01898 | Chr11: 61,982,283 - 62,032,082 |
|  | Cti_chr11_01897 | Chr11: 61,975,777 - 61,976,907 |
|  | Cti_chr11_01896 | Chr11: 61,955,438 - 61,968,296 |
|  | Cti_chr11_01895 | Chr11: 61,944,184 - 61,945,308 |
|  | Cti_chr11_01894 | Chr11: 61,895,366 - 61,896,496 |
|  | Cti_chr11_01893 | Chr11: 61,870,450 - 61,876,872 |
|  | Cti_chr3_02111 | Chr3: 88,199,603 - 88,200,739 |
|  | Cti_chr3_02112 | Chr3: 88,204,929 - 88,206,065 |
